# Supplementary material for: QKI is a critical pre-mRNA alternative splicing regulator of cardiac myofibrillogenesis and contractile function
Source: Nat Commun. 2021 Jan 4;12:89. doi: 10.1038/s41467-020-20327-5 (PMC7782589; doi:10.1038/s41467-020-20327-5)
Supplement: Supplementary file 1 — Supplementary Information [file 41467_2020_20327_MOESM1_ESM.pdf]

SUPPLEMENTARY INFORMATION

QKI is a critical pre-mRNA alternative splicing regulator of cardiac myofibrillogenesis and contractile function

Supplementary Figure and table:

Supplementary Figure 1

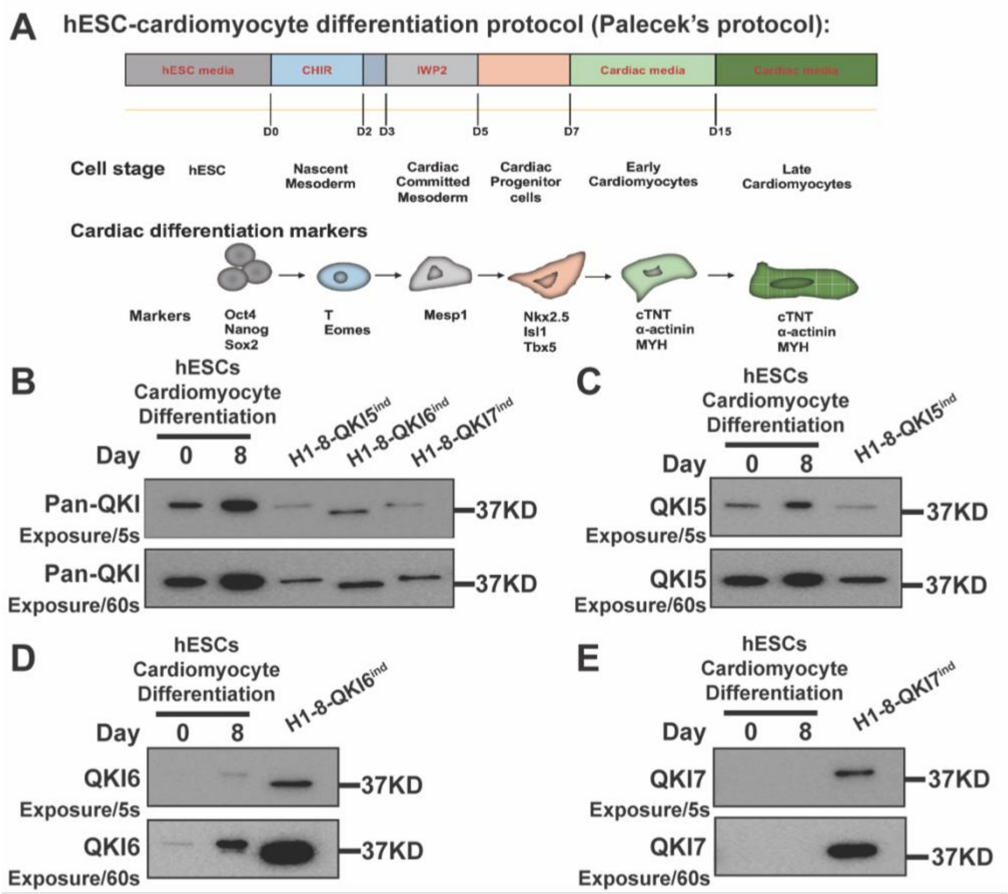

Analysis of QKI expression during hESC-to-cardiomyocyte differentiation. (A) Schematic diagram for cardiomyocyte protocol from hESCs and molecular markers for various differentiation stages; (B) Representative Western blots using anti-pan-QKI antibody (abcam and Cat#ab126742). As indicated, left two lanes are samples from Days-0 and -8 differentiated cells; the right three lanes are samples from QKI-5, QKI-6, and QKI-7 specific expressed cell lines (see Supplementary Figure 11), which is used to confirm the relative migration positions of QKIs-5, -6, and -7. Two different exposures are used to see low expression level of QKIs-6 and -7. (C) Representative Western blots using anti-QKI-5 specific antibody (Bethyl and Cat#A300-183A). (D) Representative Western blots using anti-QKI-6 antibody (Millipore and Cat#AB9906). This antibody appears to have a weak cross-reaction with QKI-5 (upper weak band, slightly above 37kD molecular weight marker, in left two lanes). Longer exposure shows a faint QKI-6 band aligned at 37kD molecular weight marker in Day-8 cardiomyocyte sample. (E) Representative Western blots using anti-QKI-7 antibody (Millipore and Cat#AB9908). Collectively, this series of Western blots conform that QKI-5 is the dominant isoform. All experiments are independently repeated at least 5 time with multiple sets of samples to ensure the reproducibility of the findings.

Supplementary Figure 2:

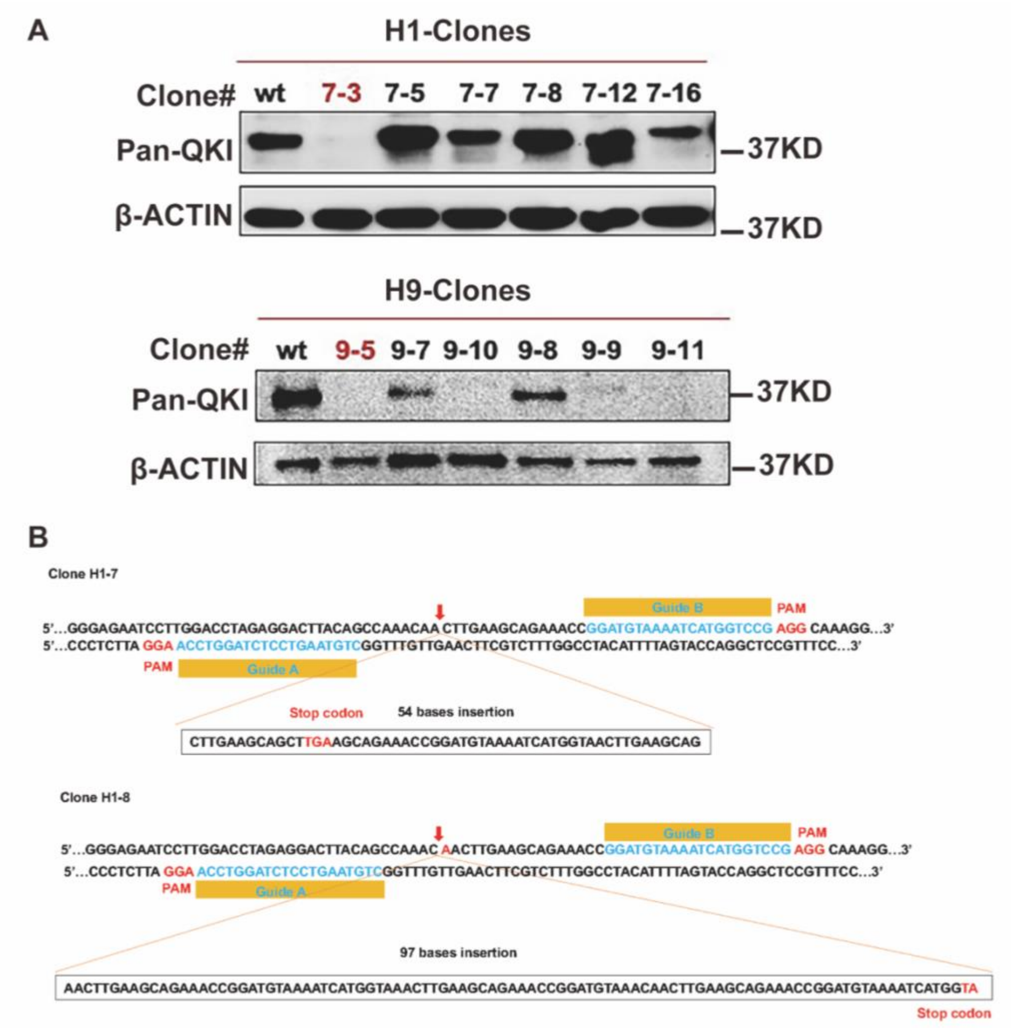

Screening for targeted clones by Western blot and sequencing analysis. (A) Representative Western blots for screening hESC-*QKI*^{del} clones in H7 and H9 hESC cell lines; The positive clones were further confirmed by at least 2 additional Western blot analyses, followed by sequencing verifications to ensure the correctly targeted clones. (B) Sequencing verification of *QKI*-targeted sites in clones H1-7 and H1-8. H1-7 showed 54 bases insertion and H1-8 showed 97 bases insertion, both resulted in a frame shift and premature STOP codon.

Supplementary Figure 3:

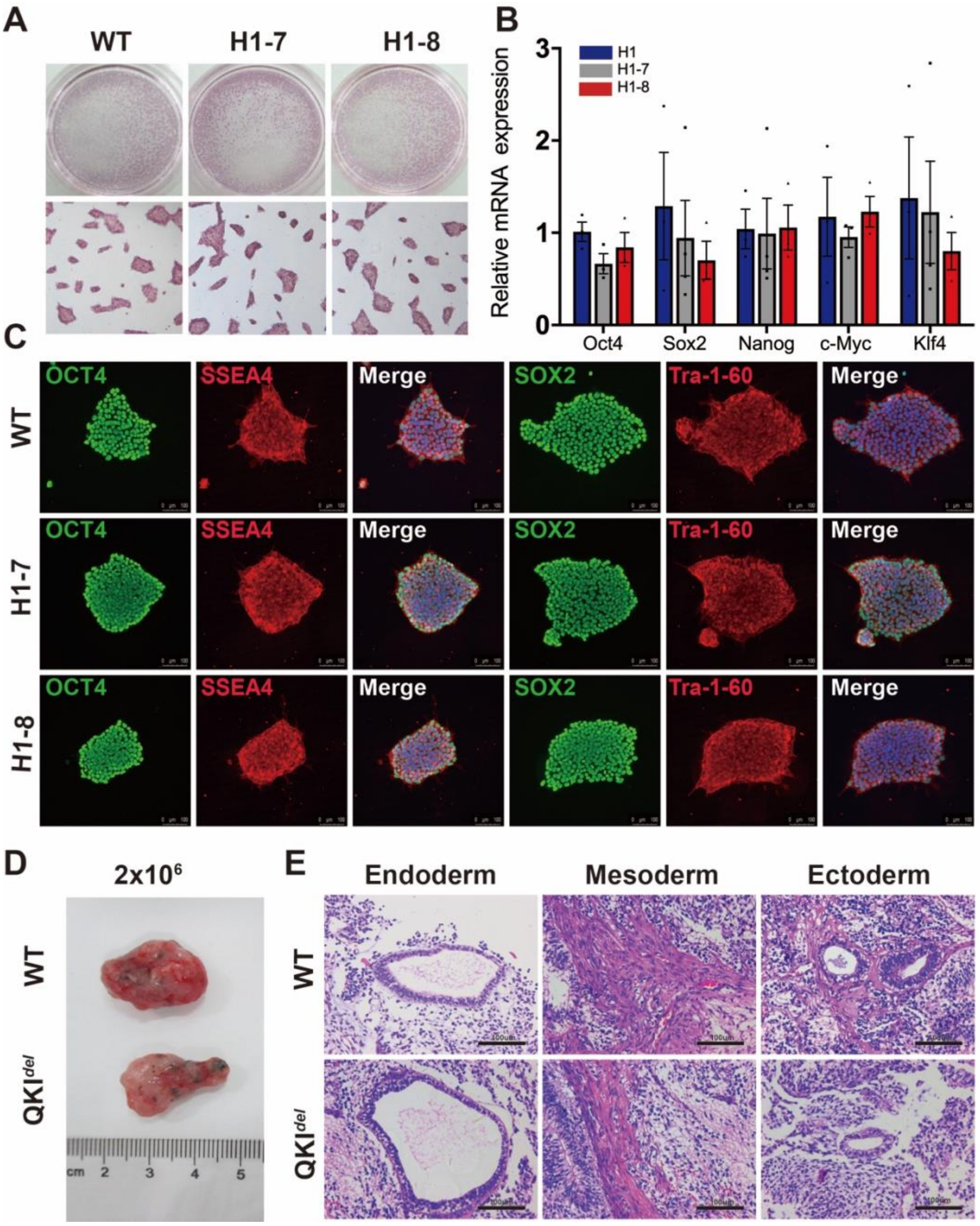

hESCs-*QKI*^{del} maintain normal self-renew and pluripotency. (A) Alkaline phosphatase (AP) staining was performed on hESCs-*QKI*^{del} (H1-7 and H1-8) and control hESCs (H1) by using the Alkaline Phosphatase Kit (Stemgent, 00-0055) according to the manufacturer's instruction. No difference was observed among the tested groups; The experiments were repeated 3 times with multiple sets of samples to ensure the reproducibility. (B) qRT-PCR analysis of the molecular markers for pluripotency, including Nanog, Oct4, Sox2, c-myc, Klf4 in hESCs-*QKI*^{del} and control hESCs. The mRNA expression levels were normalized to that of the endogenous RPL7. Data was shown as mean ± SEM, statistical significance was determined by ANOVA

test. No statistical significance was found in the expression levels among tested groups; (C) Representative images of immunofluorescence staining of the pluripotent markers OCT4, SOX2, Tra-1-60 and SSEA4 in hESCs-*QKI*^{del} and control hESCs. There is no difference observed among tested groups; Scale bar: 100μm. The experiments were independently repeated at least 3 time with multiple sets of samples to ensure the reproducibility of the finding. (D) Representative morphology teratoma formed by injecting $2x10^6$ hESCs-*QKI*^{del} and normal control hESCs (in 200 μl matrigel) into 8-week old SCID/NOD mice. Similar teratomas in size (1-1.5cm) were formed after four weeks; (E) Representative images of histological analysis of teratomas. The endodermal, mesodermal, and ectodermal layers were well formed in teratomas derived from both hESCs-*QKI*^{del} and control hESCs. Scale bar: 100μm. The experiments were independently repeated at least 3 time with multiple sets of samples to ensure the reproducibility.

Supplementary Figure 4:

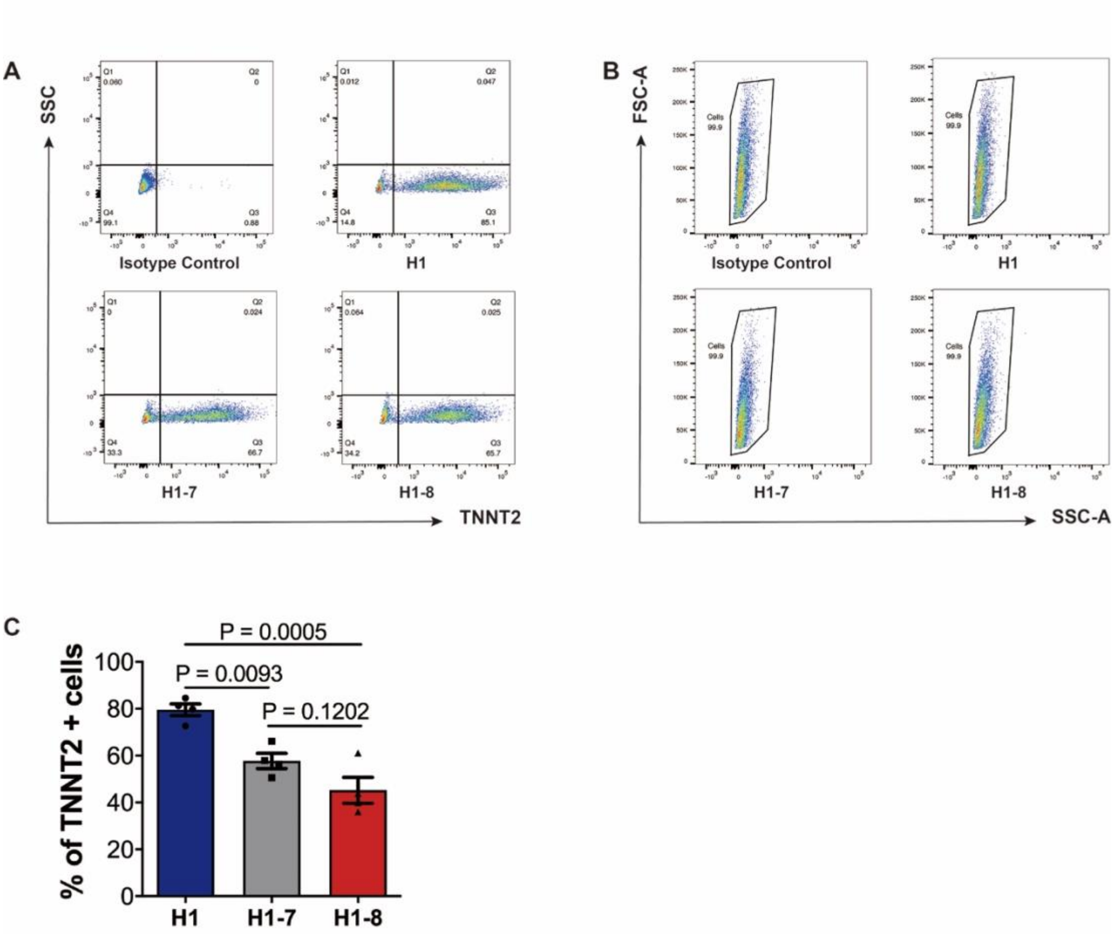

Flow cytometry analysis of TNNT2 positive cells at Day-15. (A) Representative flow cytometry analysis chart to determine the amount of TNNT2 positive cells in hESCs-*QKI*^{del} (H1-7 and H1-8) and control hESCs (H1) differentiated at Day-15. Beating hESC derived cardiomyocyte sheets were dissociated into single cells by collagenase I (1 mg/ml, Sigma) for 60 min followed by 0.25% trypsin without EDTA treatment for 10 min at 37˚C. Filter cell suspension with a 40-µm cell strainer (BD Falcon) to remove cell clumps, the cells were fixed and permeabilized using BD Cytofix /Cytoperm TM (BD 554722) for 30 min at 4°C and were incubated with PE conjugated mouse anti-human TNNT2 antibody (BD, 564767). Cells were washed twice in BD perm/wash buffer, centrifuged and resuspended in 200ul PBS. To define the threshold for positive fluorescence, the isotype control sample was incubated with PE conjugated mouse IgG k Isotype control antibody. Data were collected using FACSCalibur (BD Biosciences) and analyzed using FlowJo7.6. (B) Representative single cell gate plotted as FSC-A (forward scatter area) vs SSC-A (side scatter area). (C) A total of 10,000 gated events were counted for each marker in four independent experiments. Data was shown as mean ± SEM, statistical significance was determined by one-way ANOVA test.

Supplementary Figure 5:

A

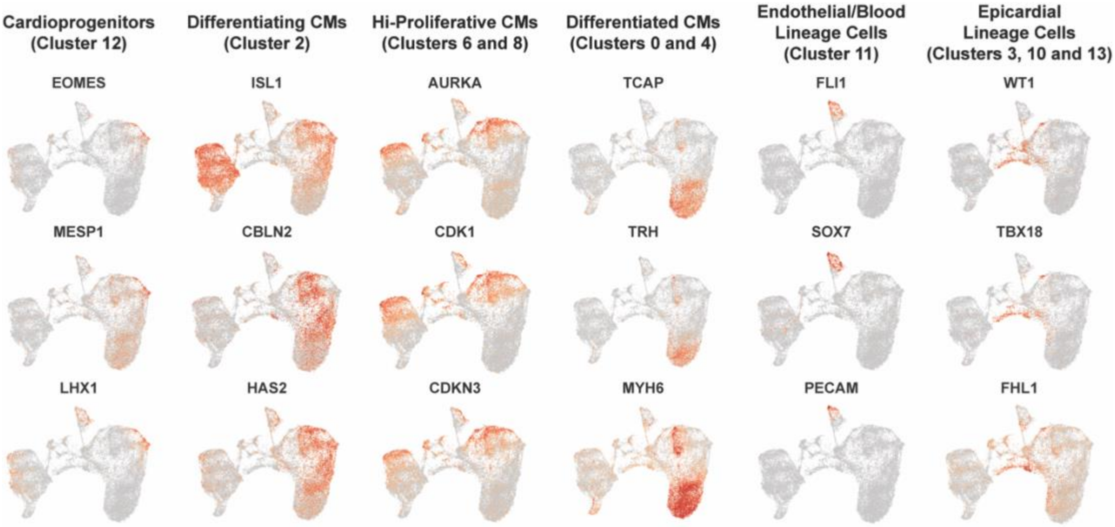

B

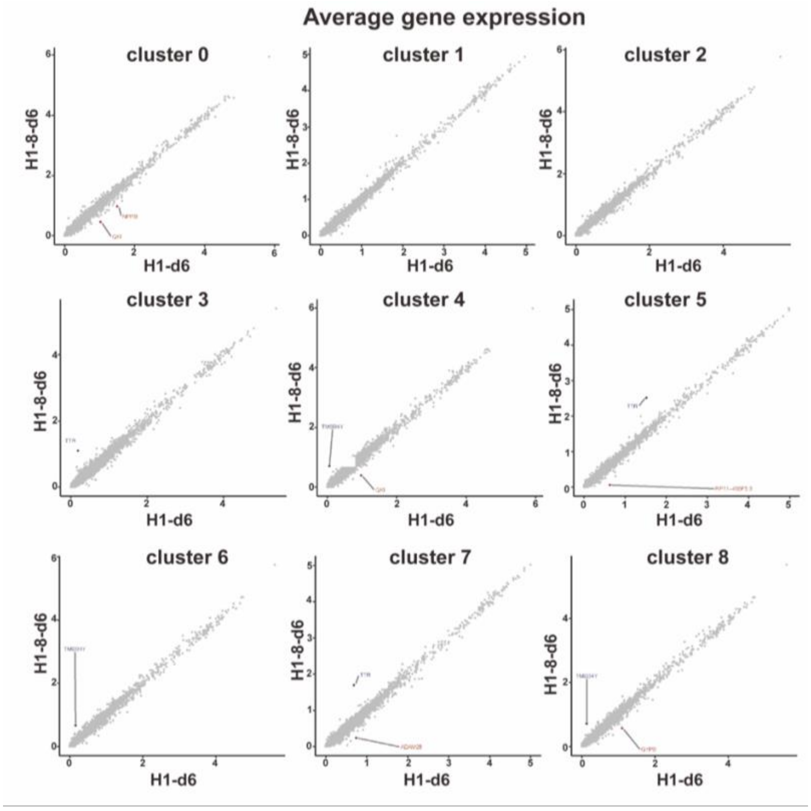

Single cell sequencing analysis of differentiated cells towards cardiomyocytes. (A) Representative expression pattern of unique functional marker genes in defining differentiation states of cardiogenic progenitors, differentiating cardiomyocytes, high proliferative cardiomyocytes, differentiated cardiomyocytes, endothelial/blood lineage cells, and epicardial lineage cells; (B) Representative scatter plots of gene expression profiles comparing major clusters of Day-6 control H1 differentiated cardiomyocytes and mutant H1-8 differentiated cells.

Supplementary Figure 6:

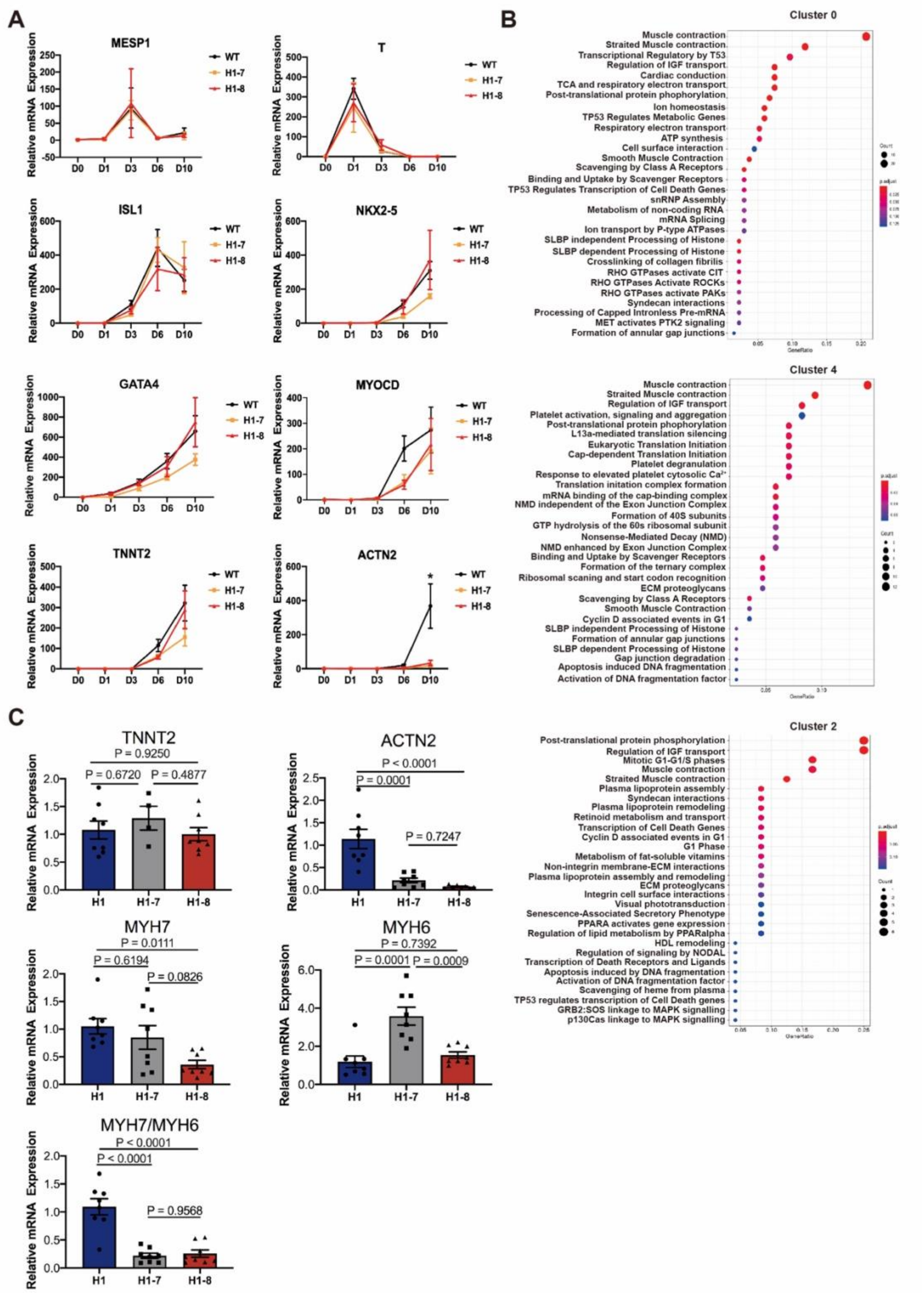

Confirmation of key molecular markers for cardiomyocyte differentiation. (A) qRT-PCR analysis was performed in differentiated cells at Days-0,-1,-3,-6, and -10, confirming the overall expression pattern of these major lineage markers was largely unchanged during the early differentiation of hESC-*QKI*^{del}; Data were shown as mean ± SEM, n=3, *P<0.0001 was determined by ANOVA test between H1 to H1-7 and H1

to H1-8, no significance was found between H1-7 and H1-8. (B) To determine the major altered biological events, Reactome Pathway Analysis (ReactomePA) [1] were performed based on the average gene expression profiles of control H1 and H1-8 mutant clusters 0, 4, and 2 at Day-15; (C) qRT-PCR analysis was performed in differentiated cells at Days-15, the reduction of *ACTN2* expression, and ratio of *MYH7*/*MYH6* confirmed the defect of cardiomyocyte maturation in hCMs-QKI^{del}. Data were shown as mean ± SEM, statistical significance was determined by one-way ANOVA test.

Supplementary Figure 7:

Total Target Sequences = 3204, Total Background Sequences = 382102

Rank	Motif	Name	P-value	log P-pvalue	q-value (Benjamini)	# Target Sequences with Motif	% of Targets Sequences with Motif	# Background Sequences with Motif	% of Background Sequences with Motif
1	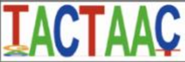	M262_0.6; RBP=QKI source=D family=KH	1e-38	-8.893e+01	0.0000	783.0	24.44%	59264.0	15.51%
2	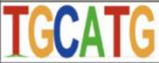	M298_0.6; RBP=A2BP1 source=D family=RRM	1e-30	-7.131e+01	0.0000	1045.0	32.62%	89921.0	23.53%
3	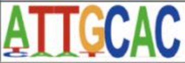	M318_0.6; RBP=SNRPA source=D family=RRM	1e-16	-3.800e+01	0.0000	1413.0	44.10%	140930.0	36.88%
4	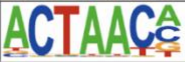	M046_0.6; RBP=QKI source=D family=KH	1e-14	-3.324e+01	0.0000	2242.0	69.98%	242414.0	63.43%
5	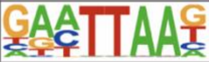	M085_0.6; RBP=ZCRB1 source=D family=CCHC ZF,RRM	1e-11	-2.703e+01	0.0000	1868.0	58.30%	199373.0	52.17%

Motif enrichment analysis of alternative splicing events. Based on RNA binding consensus sequences for known RNA binding proteins, QKI binding motif was among the topmost significantly enriched motifs in alternative splicing events. RNA-binding protein motif enrichment. We evaluated the five major types of alternatively spliced events: skipped-exons, mutually exclusive exons, retained-introns, alternative 5’ exon stop sites and alternative 3’ exon stop sites. Three sequence regions were extracted for all events found in the GRCh38v95 Ensembl annotation; These regions included the entire alternatively spliced exon and 400bp up- and down-stream of the alternatively spliced exon. In the case of mutually exclusive events, three regions were extracted for both alternatively included exons. For retained-intron events, the entire intron sequence was extracted as well as two 400bp regions extending into the surrounding exons. All extracted sequences were collected in a single FASTA file and used as the “background”, or sequences representative of areas where RNA-binding proteins may bind. A second file containing only the extracted sequences associated with the significant (FDR <=0.05) alternatively spliced events was used as the “foreground”. HOMER was then used with the arguments “-min 1 -noweight -nomotif -b” to perform motif enrichment scoring using only Homo sapiens RNA-binding protein motifs from the CISBP-RNA database [2,3]. We used HOMER (http://homer.ucsd.edu/homer/motif/) for the statistical calculation, in which motif enrichment is calculated using the cumulative binomial distribution. Significance was determined by adjusted P value (q-value, <0.05).

Supplementary Figure 8:

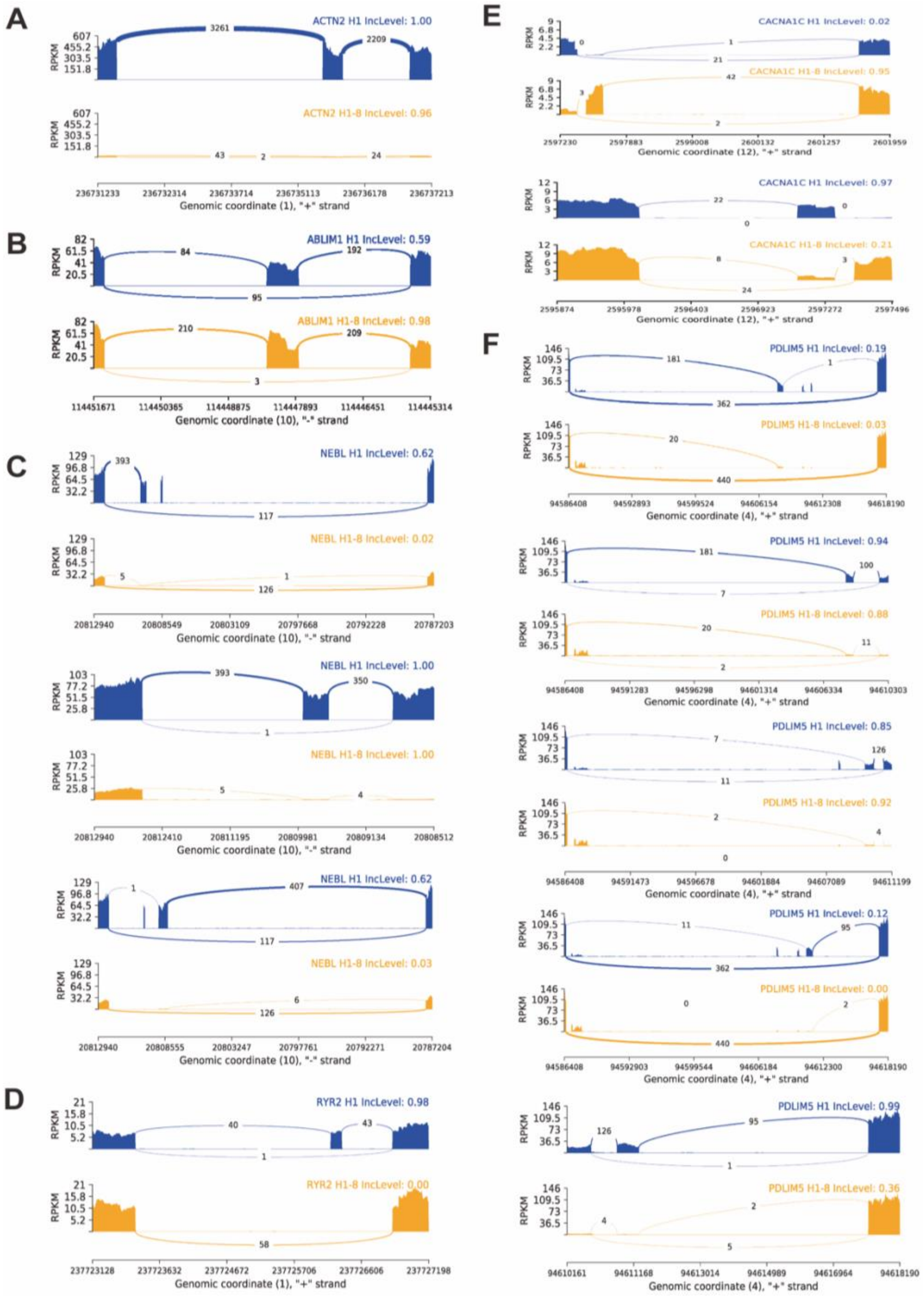

Representative sashimi plots for skipped exon events in representative genes contributing to Z-discs and calcium signaling. (A) ACTN2, (B) ABLIM1, (C) NEBL, (D) RYR2, (E) CACNA1C, and (F) PDLIM5. The sashimi plots in blue represent normal splicing events in normal control H1 cardiomyocytes, and the sashimi plots in orange represent abnormal splicing events in H1-8 mutant cardiomyocytes.

Supplementary Figure 9:

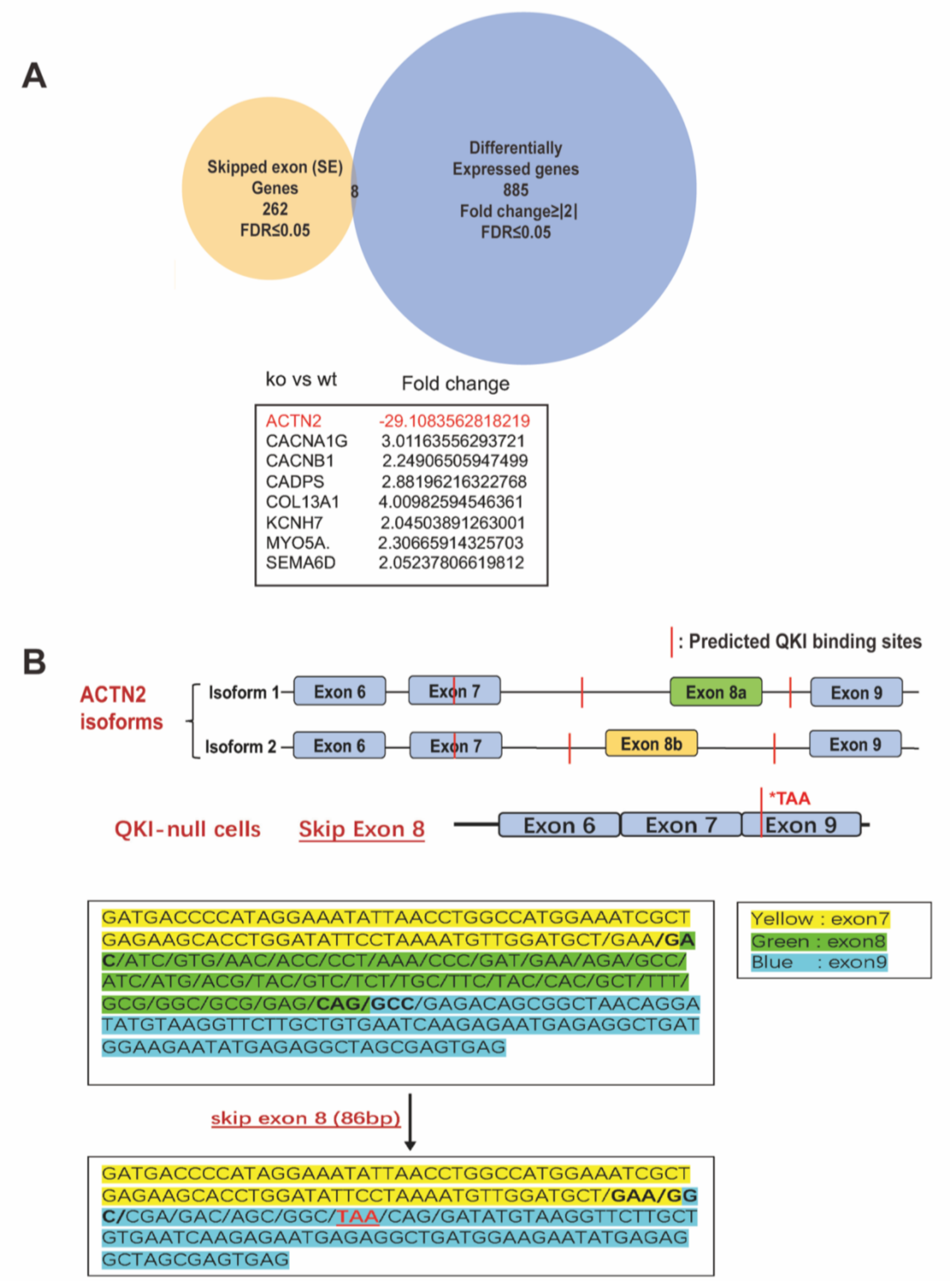

Comparison of total SE events and the differential expressed genes in Day-15 hCM-*QKI*^{del}. (A) Total of 8 genes were found to have SE events and altered expression levels in Day-15 hCM-*QKI*^{del}. ACTN2 was the most significant gene in both SE events and altered expression level; (B) Sequencing confirmation of exon 8 skipping in *ACTN2* leading to a frameshift and generating a premature STOP codon.

Supplementary Figure 10:

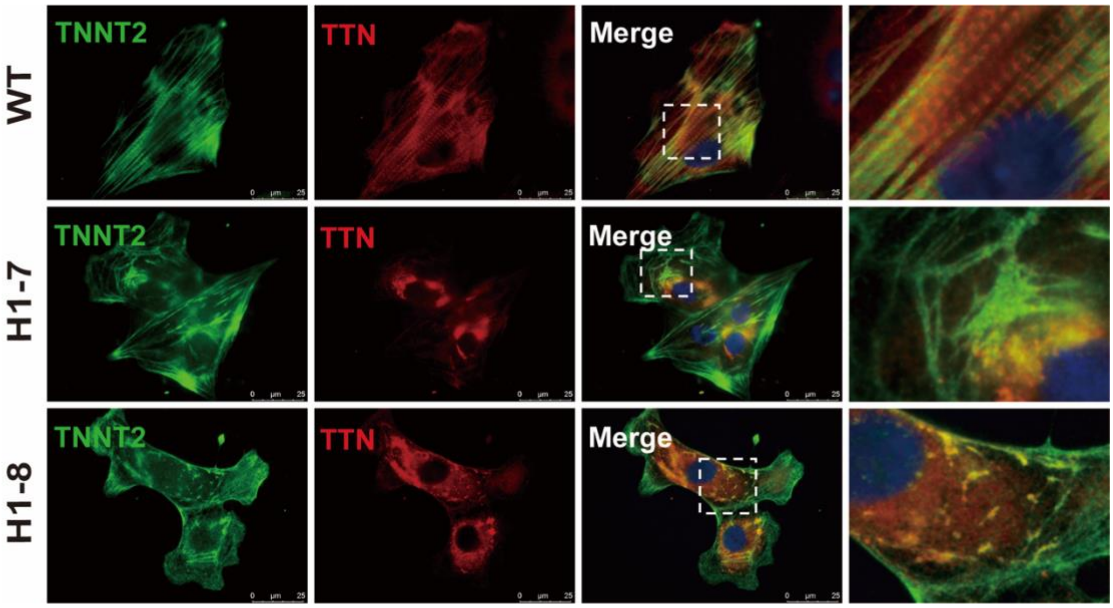

Altered myofibrillar structure in hCM-*QKI*^{del}. Representative images of immunofluorescence staining of TNNT2 (green) and TTN (red) in H1, H1-7 and H1-8 cardiomyocyte at differentiation Day-15. Scale bar: 25µm; The experiments were independently repeated at least 3 time with multiple sets of samples to ensure the reproducibility of the finding.

Supplementary Figure 11:

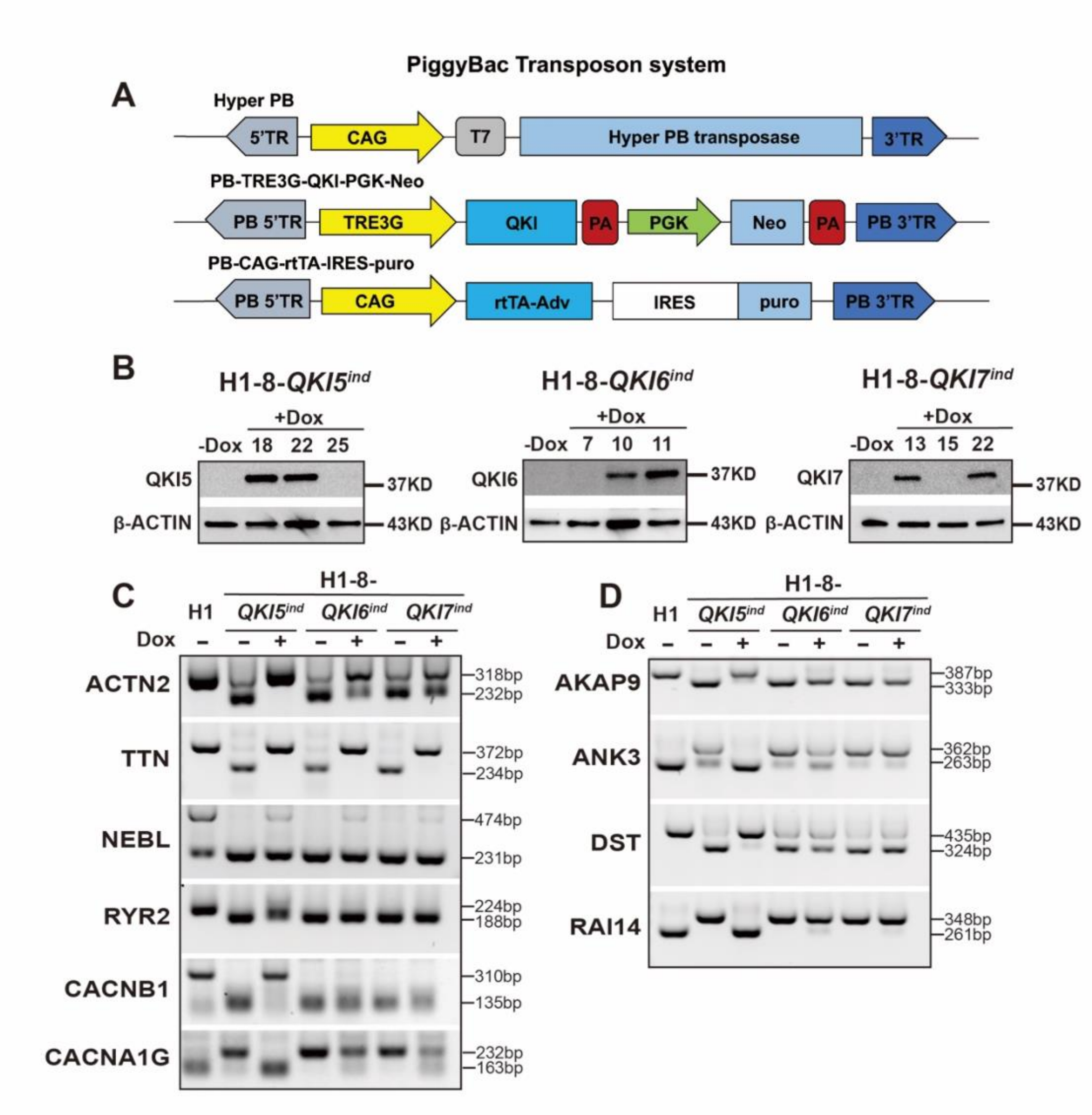

Reactivation of QKI in hESCs-*QKI*^{del} using an inducible expression system. (A) Schematic diagram of PiggyBac Transposon inducible expression system, by which *QKI* expression can be re-established in hESCs-*QKI*^{del} by doxycycline induction. cDNA fragments for *QKI5*, *QKI6*, and *QKI7* were cloned into vector PB-TRE3G-PGK-Neo, respectively; (B) Representative Western blots used for screening positive clones H1-8-*QKI5*^{ind}, H1-8-*QKI6* ^{ind}, H1-8-*QKI7* ^{ind}; (C and D) Normal alternative splicing pattern was able to achieved by reactivation of QKI5 in H1-8 at Day-15, but not by reactivation of QKI6 and QKI7. All experiments are independently repeated at least 3 time with multiple sets of samples to ensure the reproducibility of the findings.

Supplementary Figure 12:

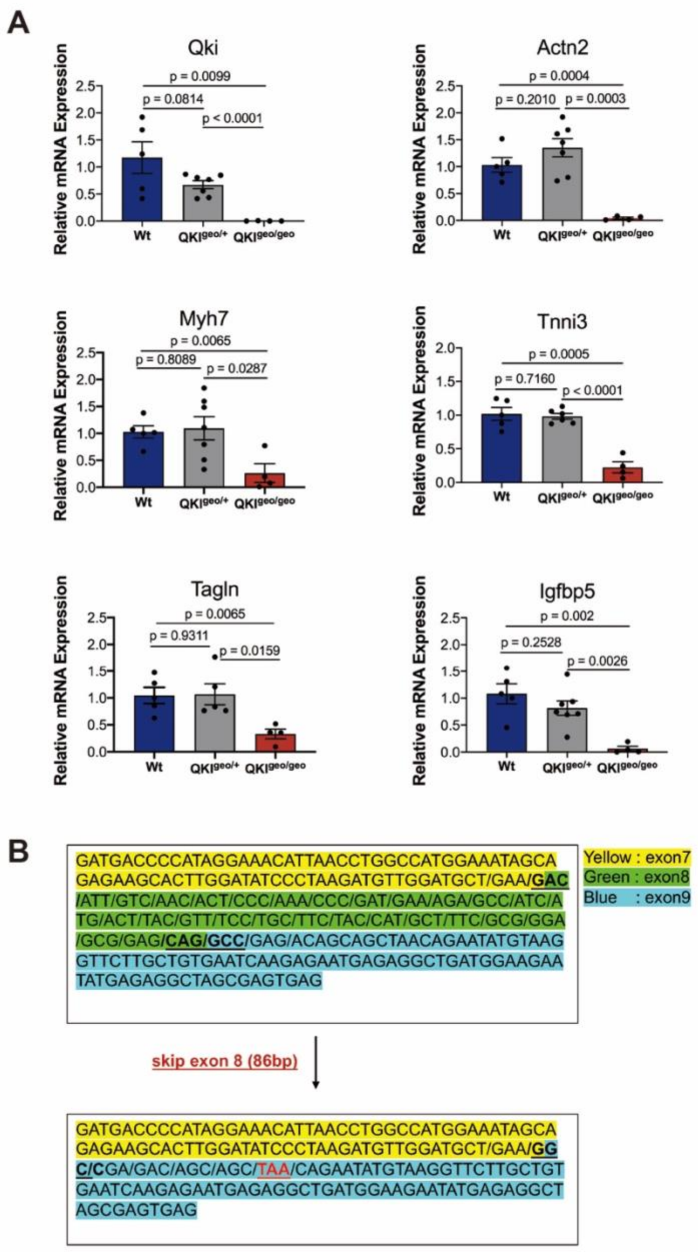

The specific function of *Qki* during mouse heart development. (A) qRT-PCR analysis to confirm a similar pattern of downregulation of altered gene expression in *Qki*$^{\beta Geo/\beta Geo}$ mouse embryonic hearts (E9.0). Data were shown as mean ± SEM, statistical significance was determined by one-way ANOVA test; (B) Sequencing analysis confirmed exon 8 of *Actn2* was skipped, which generated an identical premature STOP in *Actn2*$^{SE\text{-}8}$ in comparison to ACTN2$^{SE\text{-}8}$ identified in hCMs-*QKI*^{del}.

Supplementary Table 1

Primer sequences used for Real-Time PCR analysis and verification of alternative splicing events.

qRT-PCR primers	Forward	Reverse
human T	AAGGAGCTCACCAATGAGATG	TGACTTTGCTGAAGGAGACG
human MESP1	GAT GGA GCC AAG CCC ACC GTC	CTG CCT GAG GAG CCC AAG TGA
human ISL1	CTGTGCTGAACGAGAAGCAGC	GTAAGCCACCGTCGTGTCTCTC
human NKX2.5	GCCAAGTGTGCGTCTGCCTTTC	GCCTGCGAGAAGAGCACG
human TNNT2	GGAGGAGTCCAAACCAAAGCC	TCAAAGTCCACTCTCTCTCCATC
human OCT4/3	GCAAAGCAGAAACCCTCGTG	CACACTCGGACCACATCCTT
human SOX2	ATGGACAGTTACGCGCACAT	CGAGCTGGTCATGGAGTTGT
human NANOG	CTTCACCTATGCCTGTGATTTG	GCTGAGGTATTTCTGTCTCTG
human KLF4	GTCTCTTCGTGCACCCACTT	GGTGGCATGAGCTCTTGGTAAT
human cMYC	TACAACACCCGAGCAAGGAC	GAGGCTGCTGGTTTTCCACT
human GATA4	ACCACAAGATGAACGGCAT	CGTGGAGCTTCATGTAGAGG
human Myocardin	TTCAGAGGTAACACAGCCTCC	TGATCCTCTCTAGCGTCTGCT
human pan-QKI	ATGGTCGGGGAAATGGAAACGA	CCACAGCATCAGGCAATTCTG
human Qki5	CTGTCATGCCAAACGGAAC	GATGGACACGCATATCGTG
human Qki6	CTGTCATGCCAAACGGAAC	TTCGTTGGGAAAGCCATAC
human Qki7	CTGTCATGCCAAACGGAAC	CATGACTGGCATTTCAATCCAC
human ACTN2	CGTCGCTGACAGAGGTGC	CACCGATCATTGACATTCACAGC
human RPL7	TCGAATGGCGAGGATGGCAAG	GGCTCTACAATCCTCAGCATGTTAATCG
human MYH6	GCTGGTCACCAACAATCCCTA	CGTCAAAGGCACTATCGGTGG
human MYH7	ACTGCCGAGACCGAGTATG	GCGATCCTTGAGGTTGTAGAGC
mouse Nppa	TCCTGTGTACAGTGCGGTGTCC	CGTCTCTCAGAGGTGGGTTGAC
mouse Ankrd1	CTGAACCTGTGGATGTGCCGAG	GATCGCCAAGTGTCCTTCTAAGCATG
mouse Tagln	GGGATCGAAGCCAGTGAAGGTG	AGCCATTAGAGTCCTCTGCACTGC
mouse Myl2	AGCCAAGAAGCGGATAGAAGGCG	GCGTTGAGAATGGTCTCTTCAGGATCAG
mouse Myh7	GTATCGCATCCTGAACCCAGCAG	GCTCCAGCAGCTTCTTGAACTCC
mouse Tnni3	CAGGAGATGGAACGAGAGGCAG	CGTGAAGCTGTCGGCATAAGTC
mouse Igfbp5	AGCTACGGCGAGCAAACCAAG	TGTTCGGATTCCTGTCTCATCTCAG
mouse Actn2	CAGAACGAGGTGGAGAAGGTGAT	CGCTCATTAGCATGTTGGCGA
mouse Qki	AGCTGCTCTGCGTACACCTAC	CTCGGTCTGCGGTCACAATCC
**Primers for splicing event verification**	**Forward**	**Reverse**
human ACTN2	GCCTGACCTCATTGACTACTCAAAGC	GACAGCGGCTAACAGGATATGTAAG
human AKAP9	GATGGGCAGAGTCCTTCCAAGAAG	GGACTGTCTTGTGCTCCTTGTTCAG

human RYR2	CCTGCTATTAGATGGCAAATGGCTC	CTCTGCTTGGACAGTAGTTTATGC
human CACNA1G	CCTTCGGCAACTACGTGCTCTTC	GCCAAGCACTTCTTCCTGTCC
human CACNB1	AGTTCCAGTCTGGGAGATGTGGTG	GTAGCCCTTGAGCGACGGTC
human ABLIM1	CAAGGAATCAACATTTACCGAAAGCCAC	CATTAATTGCTCTTCTTGAAGCTGCCG
human TTN	CCTAAAGTCATAGTTGCCACACCC	GGTCTGCTGAGCATAGGATTCTTC
human NEBL	CGATCCTGTGACAGAGAGAGTGAG	TGGATGACACACTGGTCTGGTG
human ANK3	TGAAGTCGCCAATCTCCTGCTAC	GCTCAGGTTCACATTCGCATTTCTAC
human RAI14	CCTGTGAGATTGGCAGCTCTAACG	CCGATTCCTTTCCCGATAGTGGAG
human DST	AGGAAGGACAAACATGGAACTGCGT	CAGCAAACTGTGTTCGGACTCTGG
mouse Nebl	GCTTCACTCCCGTTGTGGATGATC	GCATTGACCTCATGGACGACACG
mouse Ablim1	CCGAAAACCACCCATCTACAAACAGC	CTTCATCAACTGTTCTTCTTGAAGCTGCC
mouse Akap9	GCTCGGAAAGTTCTCAGAGGGTAGAC	GAAGACAGGTCTGACTGGACTGAGC
mouse Ank3	GGCATGAGGATGTAGCTGCGTTC	GCGATGTGCAGTGGTGTATAGCC
mouse Dst	CTAGCAGACGGTGCCAGCC	GTAGTCGCAGCTTGCTTCCCTG
mouse Ral14	GTCGATTCTCTCGGACACAATGCC	CGTGGGGAGGACACATCACTC
mouse Ryr2	AATCAGGTGGCAGATGGCTCTC	CTCTGCTTAGAGAGTAGTTTGTGCC
mouse Actn2	GCCTTGGACTCTGTGCCCTCATC	CTCAGGCGTCCTGTTCTCCAGC
mouse TTN	CAGTGGTACCCAAAGTCATAGTTGCCAC	CACAGCAGCTACAACTGTTGCCAC
mouse Cacnb1	CAAGTCAGGTGACAACTCCAGTTCC	TCATAGCCCTTGAGCGACGGT
mouse Cacna1g	CCTCCACGTCATCTTGGGCT	GACTCAGACTTGGTGGCATCTCC

Supplementary References:

[1] *G Yu*, QY He*. ReactomePA: an R/Bioconductor package for reactome pathway analysis and visualization. *Molecular BioSystems* 2016, 12(2):477-479.
doi: 10.1039/C5MB00663E

[2] Heinz S, Benner C, Spann N, Bertolino E et al. Simple Combinations of Lineage-Determining Transcription Factors Prime cis-Regulatory Elements Required for Macrophage and B Cell Identities. Mol Cell 2010 May 28;38(4):576-589. PMID: 20513432

[3] Ray D, Kazan H, Cook KB, Weirauch MT, Najafabadi HS, Li X, Gueroussov S, Albu M, Zheng H, Yang A, Na H, Irimia M, Matzat LH, Dale RK, Smith SA, Yarosh CA, Kelly SM, Nabet B, Mecenas D, Li W, Laishram RS, Qiao M, Lipshitz HD, Piano F, Corbett AH, Carstens RP, Frey BJ, Anderson RA, Lynch KW, Penalva LO, Lei EP, Fraser AG, Blencowe BJ, Morris QD, Hughes TR. A compendium of RNA-binding motifs for decoding gene regulation. Nature. 2013 Jul 11;499(7457):172-7. doi: 10.1038/nature12311. PubMed PMID: 23846655.
