# Supplementary material for: QKI is a critical pre-mRNA alternative splicing regulator of cardiac myofibrillogenesis and contractile function
Source: Nat Commun. 2021 Jan 4;12:89. doi: 10.1038/s41467-020-20327-5 (PMC7782589; doi:10.1038/s41467-020-20327-5)
Supplement: Supplementary file 3 — Description of Additional Supplementary Files [file 41467_2020_20327_MOESM3_ESM.docx]

**Description of Additional Supplementary File**

**QKI is a critical pre-mRNA alternative splicing regulator of cardiac myofibrillogenesis and contractile function**

**Supplementary Movie Legends:**

**Supplementary Movie 1:** Representative movie to demonstrate the strong and synchronized spontaneous-beating activity of H1-cardiomyocyte monolayer cardiomyocyte sheet at Day-15.

**Supplementary Movie 2:** Representative movie to demonstrate the weak and asynchronized spontaneous-beating activity of H1-7-monolayer cardiomyocyte sheet at Days-15.

**Supplementary Movie 3:** Representative movie to demonstrate the weak and asynchronized spontaneous-beating activity of H1-8-monolayer cardiomyocyte sheet at Days-15.

**Supplementary Movie 4:** Representative movie to demonstrate the strong and synchronized spontaneous-beating activity of H1-cardiomyocyte monolayer cardiomyocyte sheet at Day-30.

**Supplementary Movie 5:** Representative movie to demonstrate the weak and asynchronized spontaneous beating activity of H1-7-monolayer cardiomyocyte sheet at Day-30.

**Supplementary Movie 6:** Representative movie to demonstrate the weak and asynchronized spontaneous beating activity of H1-8-monolayer cardiomyocyte sheet at Day-30.

**Supplementary Movie 7:** Representative movie to demonstrate the weak and asynchronized spontaneous beating activity of Day-15 monolayer cardiomyocyte sheet of hESCs-*QKI^del^*:*QKI5^ind^* without Dox induction.

**Supplementary Movie 8:** Representative movie to demonstrate the strong and synchronized spontaneous beating activity of Day-15 monolayer cardiomyocyte sheet of hESCs-*QKI^del^*:*QKI5^ind^* with Dox induction.

**Supplementary Movie 9:** Representative movie to demonstrate the weak and asynchronized spontaneous beating activity of Day-15 monolayer cardiomyocyte sheet of hESCs-*QKI^del^*:*QKI6^ind^* with Dox induction.

**Supplementary Movie 10:** Representative movie to demonstrate the weak and asynchronized spontaneous beating activity of Day-15 monolayer cardiomyocyte sheet of hESCs-*QKI^del^*:*QKI7^ind^* with Dox induction.
