# Supplementary material for: QKI is a critical pre-mRNA alternative splicing regulator of cardiac myofibrillogenesis and contractile function
Source: Nat Commun. 2021 Jan 4;12:89. doi: 10.1038/s41467-020-20327-5 (PMC7782589; doi:10.1038/s41467-020-20327-5)
Supplement: Supplementary file 14 — Reporting Summary [file 41467_2020_20327_MOESM14_ESM.pdf]

nature research

Corresponding author(s): Weinian Shou, Ning Sun, Guoying Huang

Last updated by author(s): Oct 15, 2020

Reporting Summary

Nature Research wishes to improve the reproducibility of the work that we publish. This form provides structure for consistency and transparency in reporting. For further information on Nature Research policies, see Authors & Referees and the Editorial Policy Checklist .

Statistics

For all statistical analyses, confirm that the following items are present in the figure legend, table legend, main text, or Methods section.

n/a	Confirmed	
☐	☒	The exact sample size (*n*) for each experimental group/condition, given as a discrete number and unit of measurement
☐	☒	A statement on whether measurements were taken from distinct samples or whether the same sample was measured repeatedly
☐	☒	The statistical test(s) used AND whether they are one- or two-sided<br>*Only common tests should be described solely by name; describe more complex techniques in the Methods section.*
☐	☒	A description of all covariates tested
☐	☒	A description of any assumptions or corrections, such as tests of normality and adjustment for multiple comparisons
☐	☒	A full description of the statistical parameters including central tendency (e.g. means) or other basic estimates (e.g. regression coefficient) AND variation (e.g. standard deviation) or associated estimates of uncertainty (e.g. confidence intervals)
☐	☒	For null hypothesis testing, the test statistic (e.g. *F*, *t*, *r*) with confidence intervals, effect sizes, degrees of freedom and *P* value noted<br>*Give P values as exact values whenever suitable.*
☒	☐	For Bayesian analysis, information on the choice of priors and Markov chain Monte Carlo settings
☒	☐	For hierarchical and complex designs, identification of the appropriate level for tests and full reporting of outcomes
☒	☐	Estimates of effect sizes (e.g. Cohen's *d*, Pearson's *r*), indicating how they were calculated

Our web collection on statistics for biologists contains articles on many of the points above.

Software and code

Policy information about availability of computer code

Data collection	CellRanger 2.1.0, R package Seurat (version 2.3.1 and version3.1.0) with Rstudio version 1.1.453 and R version 3.5.1, bamUtils (version 0.5.9), edgeR (version 3.24.3)
Data analysis	FlowJo7.6., R packages ggplot2 (http://ggplot2.org), ggrepel (https://github.com/slowkow/ggrepel), Monocle 3, rMATS (version 3.2.5), HOMER (http://homer.ucsd.edu/homer/motif/), PRISM 8.4.3, Ingenuity Pathway Analysis (IPA2020),

For manuscripts utilizing custom algorithms or software that are central to the research but not yet described in published literature, software must be made available to editors/reviewers. We strongly encourage code deposition in a community repository (e.g. GitHub). See the Nature Research guidelines for submitting code & software for further information.

Data

Policy information about availability of data

All manuscripts must include a data availability statement. This statement should provide the following information, where applicable:

- Accession codes, unique identifiers, or web links for publicly available datasets
- A list of figures that have associated raw data
- A description of any restrictions on data availability

All original data associated with all figures included in the manuscript are available from submitted SOURCE DATA file. The bulk RNA Sequence data have been deposited in Geo Database (GSE144008; link: https://www.ncbi.nlm.nih.gov/geo/query/acc.cgi?acc=GSE144008; token: wlmhyymsfjsjlcb) and single cell RNA sequence data that support the findings of this study have been deposited in Geo Database (GSE144009; link: https://www.ncbi.nlm.nih.gov/geo/query/acc.cgi?acc=GSE144009; token: wnetwoqaxzyndkx). In addition, the geneontology (GO) and Kyoto Encyclopedia of Genes and Genomes (KEGG) database used in the study are available at [http://geneontology.org/] and [https://www.genome.jp/kegg/]

Field-specific reporting

Please select the one below that is the best fit for your research. If you are not sure, read the appropriate sections before making your selection.

☒ Life sciences ☐ Behavioural & social sciences ☐ Ecological, evolutionary & environmental sciences

For a reference copy of the document with all sections, see nature.com/documents/nr-reporting-summary-flat.pdf

Life sciences study design

All studies must disclose on these points even when the disclosure is negative.

Sample size	To quantify and determine the significance of our experimental results, we used unpaired student's t-test or one-way ANOVA throughout most of our work that is based on that the data distribution followed by Gaussian distribution, which required minimal 3 samples in each experiments in order to have a statistical value. As our routine practice, most experiments were set to have at least 5 independent samples and independently repeated at least 3 times to ensure the statistical value and reproducibility. As our standard approach, 4 independent samples for each experimental group (i.e., wt vs mutant) were used in transcriptomic analysis to determine the differential expressed genes and altered biological process and pathways, which used R programming based on the hypergeometric distribution.
Data exclusions	No data was excluded from analysis, except for necessary data quality control step for initial RNA-seq data collection before .bioinoformation analysis.
Replication	All experiments described in the manuscripts were repeated at least 3 independent times with multiple samples collected independently, which did not count for failed attempt due to technique reasons and the initial test of reagents or specific methods. RNA-seq analysis was based on 4 pairs of samples independently collected to eliminate batch effect. For qRT-PCR and Western blots, 3 or more sample replica collected independently were applied in the experiments.
Randomization	Samples were randomly allocated into the study.
Blinding	For the most part of the study using mutant mouse embryos and screening of mutant cell lines, the experiments were double blinded, in which the phenotypes analysis was prior to the confirmation of their genotypes. For the most part of Western blot, qRT-PCR, PCR, immunostaining were not arranged specifically in a double blind setting, which is due to the genotypes of the samples needed to be determined, so we can have a reasonable sample size in the experiments to compare. However, in these experiments, the order of the treatments and the sample placements were not specifically arranged, so to ensure no artifact from human error.

Reporting for specific materials, systems and methods

We require information from authors about some types of materials, experimental systems and methods used in many studies. Here, indicate whether each material, system or method listed is relevant to your study. If you are not sure if a list item applies to your research, read the appropriate section before selecting a response.

Materials & experimental systems

n/a	Involved in the study	
☐	☒	Antibodies
☐	☒	Eukaryotic cell lines
☒	☐	Palaeontology
☐	☒	Animals and other organisms
☒	☐	Human research participants
☒	☐	Clinical data

Methods

n/a	Involved in the study	
☒	☐	ChIP-seq
☐	☒	Flow cytometry
☒	☐	MRI-based neuroimaging

Antibodies

Antibodies used	OCT3/4 (Stemgent, 09-0023, LOT: J16080000000002), SSEA-4 (ThermoFisher Scientific, 41-4000), SOX2 (Stemgent, 09-0024, LOT: J16020000000013), TRA-1-60 (Stemgent, 09-0010), TNNT2 (Abcam, ab45932, LOT: GR3201673-1; BD, 564767; DSHB, AB528495), ACTN2 (Sigma, A7811, LOT: 036M4861V; Clone: EA53), NEBL (ThermoFisher Scientific, PA5-53106, LOT: UG2806226), TTN (DSHB, AB528491), MF20 (DSHB, AB2147781), MYOZ2 (ThermoFisher, PA5-76946, LOT: TH2616135), TNNI3 (Santa Cruz, SC15368, LOT: D0716), Tubulin (Sigma, T6199), bACTIN (Abcam, ab8227, LOT: GR3195348-1), Pan-QKI (Abcam, ab126742, LOT:GR79080-11), QKI-5 (Bethyl, A300-183A), QKI6 (Millipore, AB9906, LOT: 3073831), and QKI7 (Millipore, AB9908, LOT:3077123), Donkey-anti-mouse IgG-594 (Thermo Fisher, A21203, LOT: 1918277), Goat-anti-Rabbit IgG-594 (Thermo Fisher, A11012, LOT: 2090526), Donkey-anti-Goat IgG-488 (Thermo Fisher, A11055, LOT: 1915848), Goat-anti-Mouse IgG-488 (Thermo Fisher, A11001, LOT: 2090562), Goat-anti-Rabbit IgG-488 (Thermo Fisher, A11008, LOT: 2051237), Goat-anti-Mouse IgG-HRP (ThermoFisher Scientific, G21040, LOT: 2043839), Mouse-anti-Rabbit IgG-HRP (Santa Cruz, SC-2357, LOT: A0318)
Validation	Mouse-anti-Rabbit-HRP (https://www.scbt.com/zh/p/mouse-anti-rabbit-igg-hrp)<br>Goat-anti-Mouse IgG-HRP (https://www.thermofisher.com/antibody/product/Goat-anti-Mouse-IgG-H-L-Cross-Adsorbed-

Secondary-Antibody-Polyclonal/G-21040)
Goat-anti-Rabbit IgG-488 (https://www.thermofisher.com/antibody/product/Goat-anti-Rabbit-IgG-H-L-Cross-Adsorbed-Secondary-Antibody-Polyclonal/A-11008)
Goat-anti-Mouse IgG-488 (https://www.thermofisher.com/antibody/product/Goat-anti-Mouse-IgG-H-L-Cross-Adsorbed-Secondary-Antibody-Polyclonal/A-11001)
Donkey-anti-Goat-488 (https://www.thermofisher.com/antibody/product/Donkey-anti-Goat-IgG-H-L-Cross-Adsorbed-Secondary-Antibody-Polyclonal/A-11055)
Goat-anti-Rabbit IgG-594 (https://www.thermofisher.com/antibody/product/Goat-anti-Rabbit-IgG-H-L-Cross-Adsorbed-Secondary-Antibody-Polyclonal/A-11012)
Donkey-anti-mouse IgG-594 (https://www.thermofisher.com/antibody/product/Donkey-anti-Mouse-IgG-H-L-Highly-Cross-Adsorbed-Secondary-Antibody-Polyclonal/A-21203)
OCT3/4 (https://www.reprocell.com/antibodies-and-staining-kits-c10/stemab-oct4-antibody-affinity-purified-rabbit-anti-mouse-human-p265)
SSEA-4 (https://www.thermofisher.com/antibody/product/SSEA4-Antibody-clone-MC813-70-Monoclonal/41-4000)
SOX2 (https://www.reprocell.com/search/sox2)
TRA-1-60 (https://www.reprocell.com/antibodies-and-staining-kits-c10/stemab-tra-1-60-antibody-purified-mouse-anti-human-p260)
TNNT2 (https://www.abcam.com/cardiac-troponin-t-antibody-ab45932.html; https://www.bdbiosciences.com/us/reagents/research/antibodies-buffers/immunology-reagents/anti-mouse-antibodies/cell-surface-antigens/pe-mouse-anti-cardiac-troponin-t-13-11/p/564767)
ACTN2 (https://www.sigmaaldrich.com/catalog/product/sigma/a7811?lang=en&region=US)
NEBL (https://www.thermofisher.com/antibody/product/NEBL-Antibody-Polyclonal/PA5-53106)
TTN (https://dshb.biology.uiowa.edu/9-D10)
MF20 (https://dshb.biology.uiowa.edu/MF-20)
MYOZ2 (https://www.thermofisher.com/antibody/product/MYOZ2-Antibody-Polyclonal/PA5-76946)
Pan-QKI (https://www.abcam.com/qki-antibody-epr7306-ab126742.html)
QKI-5 (https://www.bethyl.com/product/A300-183A/QKI+Antibody)
QKI-6 (https://www.emdmillipore.com/US/en/product/Anti-QKI-6-Antibody,MM_NF-AB9906?ReferrerURL=https%3A%2F%2Fwww.google.com%2F)
QKI-7 (https://www.emdmillipore.com/US/en/product/Anti-QKI-7-Antibody,MM_NF-AB9908?ReferrerURL=https%3A%2F%2Fwww.google.com%2F)
TNNI3 (https://www.citeab.com/antibodies/833676-sc-15368-troponin-i-antibody-h-170)
Tubulin (https://www.sigmaaldrich.com/catalog/product/sigma/t9026?lang=en&region=US&gclid=CjwKCAjw4_H6BRALEiwAvgfzq-bS6dsh6K8zk8eT83ZPT_pne8UqYPt5VmNYB0b9p8fE8jIeiaKB5ho Ct6sQAvD_BwE)

Eukaryotic cell lines

Policy information about cell lines

Cell line source(s)	H1, H7, and H9 hESC lines were purchased from WiCell
Authentication	All H1, H7, and H9 hESC lines are commonly used cell lines in stem cell research field and are approved by NIH Human Embryonic Stem Cell Registry (https://grants.nih.gov/stem_cells/registry/current.htm)
Mycoplasma contamination	All the cell lines were tested negatively for the mycoplasma contamination
Commonly misidentified lines (See ICLAC register)	None

Animals and other organisms

Policy information about studies involving animals; ARRIVE guidelines recommended for reporting animal research

Laboratory animals	Mus Musculus, C57BL/6, male and female, embryonic samples were collected from timed-mating pregnant females, adult heart samples were collected from 2-month old mice.
Wild animals	The study did not involve wild animals.
Field-collected samples	The study did not involve field-collected samples
Ethics oversight	Indiana University School of Medicine IACUC approved the study protocol.

Note that full information on the approval of the study protocol must also be provided in the manuscript.

Flow Cytometry

Plots

Confirm that:

- [x] The axis labels state the marker and fluorochrome used (e.g. CD4-FITC).
- [x] The axis scales are clearly visible. Include numbers along axes only for bottom left plot of group (a 'group' is an analysis of identical markers).
- [x] All plots are contour plots with outliers or pseudocolor plots.
- [x] A numerical value for number of cells or percentage (with statistics) is provided.

Methodology

Sample preparation	Beating hESC derived cardiomyocyte sheets were dissociated into single cells by collagenase Ⅰ (1 mg/ml, Sigma) for 60 min followed by 0.25% trypsin without EDTA treatment for 10 min at 37˚C. Filter cell suspension with a 40-µm cell strainer (BD Falcon) to remove cell clumps, the cells were fixed and permeabilized using BD Cytofix /Cytoperm TM (BD 554722) for 30 min at 4˚C and were incubated with PE conjugated mouse anti-human TNNT2 antibody (BD, 564767).
Instrument	FACSCalibur (BD Biosciences)
Software	FlowJo7.6
Cell population abundance	A total of 10,000 gated events were counted for each marker in three independent experiments.
Gating strategy	TNNT2 positive cells were identified by the expression of TNNT2.

- [x] Tick this box to confirm that a figure exemplifying the gating strategy is provided in the Supplementary Information.
